# Maximizing Biocatalytic Cyclohexane Hydroxylation by Modulating Cytochrome P450 Monooxygenase Expression in *P. taiwanensis* VLB120

**DOI:** 10.3389/fbioe.2020.00140

**Published:** 2020-02-27

**Authors:** Lisa Schäfer, Rohan Karande, Bruno Bühler

**Affiliations:** Department of Solar Materials, Helmholtz-Centre for Environmental Research—UFZ, Leipzig, Germany

**Keywords:** whole-cell biocatalysis, *Pseudomonas*, CYP450 monooxygenase, cyclohexane hydroxylation, pSEVA

## Abstract

Cytochrome P450 monooxygenases (Cyps) effectively catalyze the regiospecific oxyfunctionalization of inert C–H bonds under mild conditions. Due to their cofactor dependency and instability in isolated form, oxygenases are preferably applied in living microbial cells with *Pseudomonas* strains constituting potent host organisms for Cyps. This study presents a holistic genetic engineering approach, considering gene dosage, transcriptional, and translational levels, to engineer an effective Cyp-based whole-cell biocatalyst, building on recombinant *Pseudomonas taiwanensis* VLB120 for cyclohexane hydroxylation. A *lac*-based regulation system turned out to be favorable in terms of orthogonality to the host regulatory network and enabled a remarkable specific whole-cell activity of 34 U g_CDW_^–1^. The evaluation of different ribosomal binding sites (RBSs) revealed that a moderate translation rate was favorable in terms of the specific activity. An increase in gene dosage did only slightly elevate the hydroxylation activity, but severely impaired growth and resulted in a large fraction of inactive Cyp. Finally, the introduction of a terminator reduced leakiness. The optimized strain *P. taiwanensis* VLB120 pSEVA_Cyp allowed for a hydroxylation activity of 55 U g_CDW_^–1^. Applying 5 mM cyclohexane, molar conversion and biomass-specific yields of 82.5% and 2.46 mmol_cyclohexanol_ g_biomass_^–1^ were achieved, respectively. The strain now serves as a platform to design *in vivo* cascades and bioprocesses for the production of polymer building blocks such as ε-caprolactone.

## Introduction

Realizing aerobic oxidation of thermodynamically stable and kinetically inert C–H bonds in cyclohexane under sustainable and environmentally safe conditions remains a major challenge in current academic and industrial research (Schuchardt et al., 2001; Cavani and Teles, 2009). Industrial-scale C6-monomer production for, e.g., Nylon 6 and Nylon 66, typically is based on the so-called liquid-phase cyclohexane oxidation (Bellussi and Perego, 2000), involving Co/Mn carboxylate salts as homogenous catalysts applied at 140–160°C and 7–20 atm with air as the oxidant (Musser, 2000). In the current industrial process, this initial cyclohexane oxyfunctionalization step, with a low yield of 6–8% for KA oil (K: cyclohexanone, A: cyclohexanol) and selectivity of 80–90% (Fischer et al., 2010), is most critical regarding economical and ecological process efficiency. Although substantial research effort has been devoted to developing novel chemical catalysts, the control of selectivity with increased conversion continues to be challenging (Schuchardt et al., 2001). Owing to the high demand, but low price, alternative production pathways need to be developed for an economical viable production process (Schuchardt et al., 1993; Van Beilen et al., 2003; Weissermel and Arpe, 2003).

With their high selectivity and catalytic effectiveness, biocatalysts often constitute a promising alternative to chemical catalysts. This especially holds true for O_2_-mediated oxyfunctionalizations, which can be realized by means of oxygenases under environmentally benign non-toxic operation conditions (Leak et al., 2009). Especially the versatile cytochrome P450 monooxygenases (Cyps), which have been employed to produce high-value compounds, constitute a promising group of enzymes (Urlacher and Schmid, 2006; Julsing et al., 2008). Due to their cofactor dependency and instability in isolated form, oxygenases are preferably applied in whole microbial cells (Schrewe et al., 2013). Recently, a class I cytochrome P450 monooxygenase (Cyp)-based whole-cell biocatalyst has been reported to perform selective cycloalkane (C5–C8) oxyfunctionalization under ambient conditions (Salamanca et al., 2015; Karande et al., 2016). To this end, respective genes have been isolated from *Acidovorax* CHX100 and functionally expressed in *Pseudomonas taiwanensis* VLB120, enabling a specific whole-cell activity of 20 U g_CDW_^–1^ for cyclohexane oxidation. This Cyp system has also been integrated into an enzyme cascade enabling the *in vivo* synthesis of lactones from cycloalkanes at specific rates of 20–22 U g_CDW_^–1^. Thereby, the Cyp activity was rate-limiting and thus constitutes the primary hurdle for establishing a viable process based on this biocatalytic approach.

The increase of gene expression levels constitutes a major strategy to improve enzyme activities *in vivo*. To this end, different approaches are followed, such as gene dosage increase by the use of vectors with high copy number (Ajikumar et al., 2010) or by integrating multiple gene copies into the genome, promoter engineering (Alper et al., 2005; Xu et al., 2013) to optimize gene transcript levels, and ribosomal binding site (RBS) engineering to optimize translation levels (Jeschek et al., 2017). In previous work, the expression plasmid pCom10, enabling expression under the control of the *alk* regulatory system from *Pseudomonas putida* GPo1, was applied for functional Cyp gene expression in *P. taiwanensis* VLB120 (Karande et al., 2016). This strain constitutes a highly interesting host strain as it can tolerate high solvent and thus substrate and product levels and provides a high metabolic capacity to support oxygenase biocatalysis also at high cell densities (Kuhn et al., 2012; Volmer et al., 2014, 2017, 2019). However, catabolite repression by glucose constitutes a major disadvantage of the pCom10 system in *Pseudomonas*, necessitating the use of a more expensive carbon and energy source such as citrate (Staijen et al., 1999). Additionally, inducers of this system such as dicyclopropylketone (DCPK) are volatile complicating its application on an industrial scale.

**FIGURE 1:**
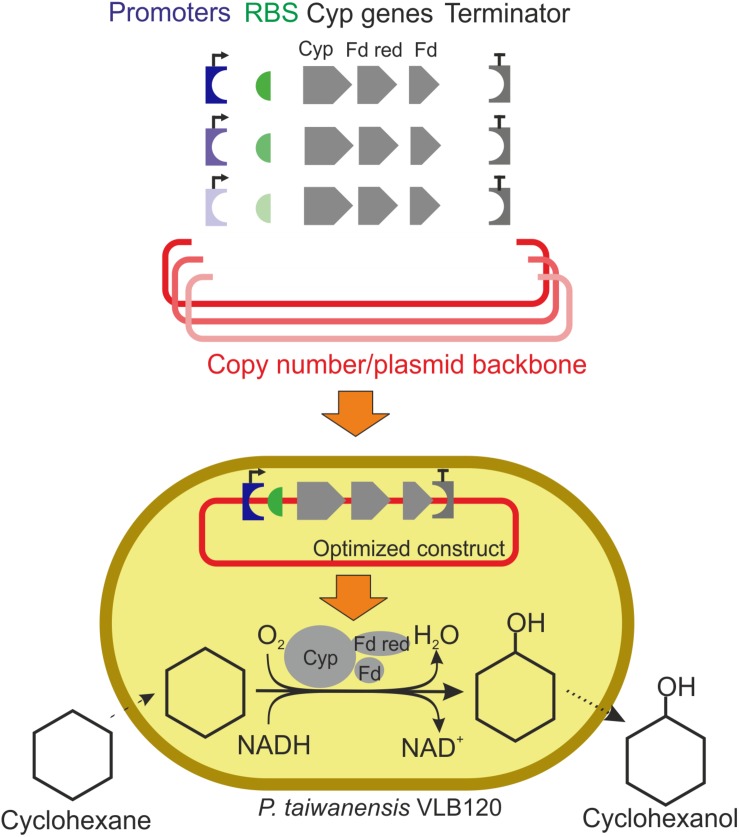
Schematic workflow of expression vector engineering to enhance cytochrome P450 monooxygenase (Cyp) gene expression and cyclohexane oxidation rates. The Cyp genes encode the three components of this enzyme system: the oxygenase (Cyp), the ferredoxin reductase (Fd red), and the ferredoxin (Fd). Plasmid backbones, promoters, RBSs, and a terminator were assembled in different constellations. Varied components are shown in colors with different intensities. Specific whole-cell activities and growth rates of resulting strains were evaluated. Specific activities are calculated based on the amount of cyclohexanol formed.

In this study, we set out to improve Cyp activities in *P. taiwanensis* VLB120 by tackling the challenges imposed by the expression vector pCom10_Cyp via a holistic approach involving transcriptional, translational, as well as gene dosage engineering (Figure 1). For this purpose, we made use of the Standard European Vector Architecture (SEVA) system (Ellis et al., 2011; Silva-Rocha et al., 2013; Martínez-García et al., 2015). Different promotor systems, RBSs, and origins of replication were evaluated by means of four readouts: growth rate, total Cyp amount in the cell, active Cyp content, and specific hydroxylation activity.

## Materials and Methods

### Bacterial Strains, Plasmids, Media, and Chemicals

Microbial strains and plasmids used in this work are listed in Table 1. Cells were grown in lysogeny broth medium (Sambrook and Russell, 2001) or M9^∗^ medium (Panke et al., 1999) with a pH of 7.2 supplemented with 0.5% (w/v) glucose or citrate as sole carbon source and kanamycin (50 μg mL^–1^) for plasmid selection. Unless stated otherwise, all chemicals were purchased from Sigma–Aldrich (Steinheim, Germany) or Carl Roth (Karlsruhe, Germany) in the highest purity available and used without further purifications.

**TABLE 1:** Strains and plasmids used in this study.

**Strain**	**Characteristics**
*E. coli* DH5α^1^	*sup*E44Δ*lacU*169(Φ80*lacZ*ΔM15) *hsd*R17 *rec*A1 *end*A1 *gyr*A96 *thi-*1 *rel*A1
*P. taiwanensis* VLB120^2^	Solvent tolerant, styrene degrading bacterium, isolated from forest soil
**Plasmids**	
pCom10_lac_Cyp	pRO1600 and ColE1ori, *lac*-regulatory system (*lacI*, *PlacUV5*), Km^R^, pCom10 RBS, Cyp genes from *Acidovorax* sp.
pCom10_tac_Cyp	pRO1600 and ColE1ori, *lac*-regulatory system (*lacI^*q*^, Ptac*), Km^R^, pCom10 RBS, Cyp genes from *Acidovorax* sp.
pSEVA244_BB32_Cyp	pRO1600 and ColE1ori, *lac*-regulatory system (*lacI^*q*^, Ptrc*), BBa_B0032 RBS, Cyp genes from *Acidovorax* sp.
pSEVA244_BB34_Cyp	pRO1600 and ColE1ori, *lac*-regulatory system (*lacI^*q*^, Ptrc*), BBa_B0034 RBS, Cyp genes from *Acidovorax* sp.
pSEVA244_RBS* _Cyp	pRO1600 and ColE1ori, *lac*-regulatory system (*lacI^*q*^, Ptrc*), RBS*, Cyp genes from *Acidovorax* sp.
pSEVA254_BB32_Cyp	RSF1010 ori, *lac*-regulatory system (*lacI^*q*^, Ptrc*), BBa_B0032 RBS, Cyp genes from *Acidovorax* sp.
pSEVA254_BB34_Cyp	RSF1010 ori, *lac*-regulatory system (*lacI^*q*^, Ptrc*), BBa_B0034 RBS, Cyp genes from *Acidovorax* sp.
pSEVA254_RBS* _Cyp	RSF1010 ori, *lac*-regulatory system (*lacI^*q*^, Ptrc*), RBS*, Cyp genes from *Acidovorax* sp.
pSEVA244_T_BB34_Cyp	pRO1600 and ColE1ori, *lac*-regulatory system (*lacI^*q*^, Ptrc*), BBa_B0015 terminator, BBa_B0034 RBS, Cyp genes from *Acidovorax* sp.
pSEVA244_T_RBS* _Cyp = pSEVA_Cyp	RSF1010 ori, *lac*-regulatory system (*lacI^*q*^, Ptac*), RBS*, Cyp genes from *Acidovorax* sp.
pCom10_Cyp^3^	pRO1600 and ColE1 ori, *alk*-regulatory system (*alkS, PalkB*), Km^R^, pCom10 RBS, Cyp genes from *Acidovorax* sp.

*^1^Hanahan, 1983. ^2^Köhler et al., 2013. ^3^Karande et al., 2016. If not stated otherwise, the plasmids and strains were constructed in this study.*

### Molecular Biology Methods

The preparation of electrocompetent *Pseudomonas* cells was performed according to Choi and Schweizer (2006), and the vectors were introduced by electroporation (2500 V, Eppendorf Eporator^®^, Hamburg, Germany). DNA manipulation methods and agarose gel electrophoresis were performed as described by Sambrook and Russell (2001). Enzymes (Phusion High-Fidelity Polymerase, T5 exonuclease, Taq ligase, restriction enzymes, Fast Alkaline Phosphatase) and buffers were purchased from Thermo Scientific Molecular Biology (St. Leon-Rot, Germany) or New England Biolabs (Frankfurt/Main, Germany) and oligonucleotides from Eurofins Genomics (Ebersberg, Germany). Plasmids were isolated using the peqGOLD plasmid Miniprep Kit I from peqLab (Erlangen, Germany) and purified via NucleoSpin Gel and PCR Clean-up from Macherey–Nagel (Düren, Germany) according to supplier protocols. The Gibson Master Mix was prepared according to Gibson et al. (2009). For detailed information, see Supplementary Table S1 and Supplementary Figure S1.

### Growth of Bacterial Cultures

Cultivations were carried out at 30 °C and 200 rpm in a Multitron shaker (Infors, Bottmingen, Switzerland). Microorganisms were inoculated from a 10% glycerol stock in a 10 mL LB pre-culture for ca. 20 h, from which a 10 mL M9^∗^ pre-culture (1% v/v) was inoculated and incubated for another 12–16 h. This culture was used to inoculate a 50 mL M9^∗^ main culture to a starting OD of 0.2 or 0.4 (only for chapter transcriptional engineering). Heterologous gene expression was induced with 1 mM isopropyl β-D-1-thiogalactopyranoside (IPTG) for *lac*-based systems or 0.025% (v/v) DCPK for the *alk*-based system after 4 or 2.5 h (only for chapter transcriptional engineering) of cultivation. Incubation was continued for another 4–6 h, and cells were harvested for SDS-PAGE and CO difference spectra analyses and/or for resting cell assays.

### Resting Cell Assays and Bioconversion Experiments

Cells were harvested by centrifugation and resuspended to a target cell concentration (as indicated) in 100 mM potassium phosphate buffer (pH 7.4) supplemented with 1% (w/v) glucose or citrate as the source for energy and reduction equivalents. For the determination of specific whole-cell activities, the cells were diluted to two different cell concentrations, i.e., 0.2 and 0.5 g_CDW_ L^–1^. In all assay setups, both gave comparable activities, which thus were averaged. The cells were transferred to baffled Erlenmeyer flasks (100 mL) or Pyrex tubes and equilibrated at 30°C for 10 min before an aqueous phase equivalent of 10 mM pure cyclohexane was added resulting in a final aqueous concentration of ∼180 μM (the major part of cyclohexane resided in the gas phase). Incubation was continued for 10 min, when the reaction was stopped. The liquid sample (1 mL) was extracted with ice-cold diethyl ether (Et2O) (1 mL) for GC analysis containing 0.2 mM n-decane as an internal standard. After 2 min extraction by vortexing and centrifugation, the organic phase was dried over anhydrous Na_2_SO_4_ before it was transferred to a GC vial for analysis. The specific cyclohexane hydroxylation activity was calculated based on the formed cyclohexanol amount within 10 min of reaction per g cell dry weight (1 U = 1 μmol cyclohexanol per min).

For the comparison of pSEVA_Cyp and pCom10_Cyp, 250 mL baffled Erlenmeyer flasks (300 mL total volume) were used applying a liquid volume of 40 mL with a cell concentration of 1.5 g L^–1^. The caps contained a septum composed of Teflon facing the inner side of the flask and silicon facing outwards. The reaction was started by adding a cyclohexane concentration of 5 mM (referred to in the aqueous phase). At each sampling point, 1.5 mL liquid volume was removed with a syringe, and gas-phase samples were taken for O_2_ quantification. One mL of the liquid sample was extracted with 1 mL of diethyl ether for GC analysis. The substrate cyclohexane as well as cyclohexanol and the overoxidation product cyclohexanone were quantified. The conversion refers to ratio of product amount (cyclohexanol and cyclohexanone) to the known added substrate amount. The selectivity expresses the fraction of cyclohexanol of the total product amount. The total turnover number (TTN) relates the produced amount of cyclohexanol to the active Cyp amount within the cells determined by CO difference spectra. The yield was calculated based on the formed cyclohexanol amount per g cell dry weight.

### CO Difference Spectra

Active amount of Cyp in whole cells was quantified by means of CO difference spectra previously described (Cornelissen et al., 2011, 2012). Cells were harvested and resuspended in 100 mM potassium phosphate buffer (pH = 7.4) containing 1% (w/v) glucose to obtain an OD_450_ of 15 in a volume of 0.9 mL. This cell suspension was transferred to a plastic cuvette and supplemented with 100 μL of fresh sodium dithionite solution (15 mg mL^–1^). The baseline was recorded with a UV–visible spectrophotometer (Varian, Type CARY 300, Palo Alto, CA, United States). Then, the sample was gassed with carbon monoxide (Linde AG, Munich, Germany) for 1 min and a CO difference spectrum was recorded between 350 and 600 nm. The Cyp-concentration was calculated using a molar extinction coefficient of 91 mM^–1^ cm^–1^ between 450 and 490 nm (Omura and Sato, 1964).

### Analytical Methods

Biomass concentrations were detected as the optical density at a wavelength of 450 nm using a Libra S11 spectrophotometer (Biochrom, Cambridge, United Kingdom). One OD_450_ unit corresponds to 0.186 g_CDW_ L^–1^ (Halan et al., 2010).

Proteins were analyzed via SDS-PAGE according to Laemmli (Laemmli, 1970), loading 30 μg of total protein per lane. Concentrations of substrates and products were determined by a GC system (Trace 1310, Thermo Scientific, Waltham, MA, United States) equipped with a flame ionization detector and a TR-5MS GC Column (dimensions: 15 m length, 0.25 mm inner diameter, Thermo Scientific) and operated with molecular nitrogen as carrier gas, 1 μL injection volume, and splitless injection mode. The temperature profile setting was as follows: 40°C (3 min), 40–170°C (15°C min^–1^), 170–300°C (100°C min^–1^). Products were quantified based on calibration curves obtained with commercially available standards. An exemplary chromatogram is given in Supplementary Figure S2.

## Results

In previous work, Karande et al. (2016) demonstrated that the Cyp genes from *Acidovorax* sp. CHX100 can be expressed in *P. taiwanensis* VLB120 under the control of the *alk* regulatory system from *P. putida* GPo1, enabling a resting cell activity of 20 U g_CDW_^–1^. The applicability of the constructed pCom10_Cyp expression vector in *Pseudomonas* is limited due to catabolite repression by glucose necessitating the use of more expensive carbon sources, the volatile nature of inducers, and the moderate cyclohexane oxidation activity achieved. To overcome these limitations, we followed an integrated and combinatorial approach involving transcription, translation, and gene dosage engineering based on the SEVA system (Silva-Rocha et al., 2013).

### Transcriptional (Promoter) Engineering for Efficient Cyp Gene Expression

**FIGURE 2:**
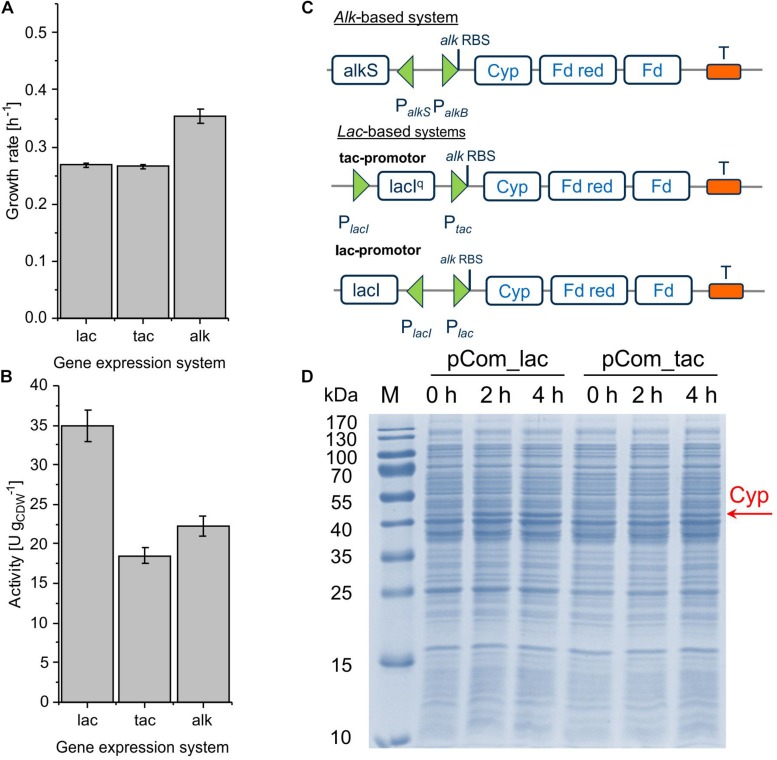
Growth rate **(A)** and specific cyclohexane hydroxylation activity **(B)** of *P. taiwanensis* VLB120 harboring pCom10_lac_Cyp, pCom10_tac_Cyp, or pCom10_Cyp after 4 h of induction. Cells were cultivated in M9^∗^ medium with 0.5% (w/v) glucose (*lac* and *tac*) or citrate (*alk*) and resuspended to biomass concentrations of 0.2 and 1.0 g_CDW_ L**^–^**^1^ in Kpi buffer supplemented with 1% (w/v) glucose or citrate, respectively. Reactions were started by adding 10 μL pure cyclohexane (180 μM in aq. phase) and stopped after 10 min by quenching with diethyl ether (see section “Materials and Methods” for details). The bars represent average values. Standard deviations of two independent biological replicates are given. The average experimental errors over all measurements for the growth rate and activity are 2.1 and 5.5%, respectively. Panel **C** illustrates the constructs applied (Cyp: Cytochrome P450 monooxygenase, Fd red: Ferredoxin reductase, Fd: ferredoxin). Panel **D** shows the SDS-PAGE analysis of the different strains 0, 2, and 4 h after induction, with the band at 47.4 kDa representing Cyp.

The alteration of gene transcript levels via promoter engineering constitutes the most common strategy for the fine-tuning of expression levels (Jeschek et al., 2017). In this work, *lac*-based regulation systems (Lindmeyer et al., 2015) were tested as alternatives to the *alk* regulatory system (Figure 2C). The vector backbone was kept the same for better comparison. Resulting strains showed decent Cyp activity, with pCom10_lac_Cyp effectuating a clearly higher specific activity of 35.0 ± 1.9 U g_CDW_^–1^ than the original construct with 22.2 ± 1.9 U g_CDW_^–1^ and pCom10_tac_Cyp with 18.5 ± 1.0 U g_CDW_^–1^ (Figure 2B and Supplementary Table S2). The strong LacI^q^ repressor present in pCom10_tac_Cyp constitutes a possible reason for the lower Cyp expression level obtained with this vector (Figure 2D). All three stains exhibited similar Cyp-specific turnover numbers indicating that the active Cyp amount in the cells limited the cell-specific activity (Table 2). However, cells bearing the *lac*-based constructs exhibited severely hampered growth, which was in contrast to cells harboring the original *alk*-based vector (Figure 2A). To profit from the higher whole-cell activity achieved with pCom10_lac_Cyp and cheap glucose as a growth substrate, the growth issue needs to be addressed. To this end, we used vector parts from the SEVA platform (Silva-Rocha et al., 2013) for further engineering (see below).

**TABLE 2:** Specific whole-cell activities, Cyp concentrations, and Cyp turnover numbers obtained with the different expression vectors constructed.

**Plasmid backbone**	**RBS**	**Activity (U g_CDW_^–1^)**	**Active Cyp concentration^1^ (nmol g_CDW_^–1^)**	**Turnover number (mol min^–1^ mol^–1^)**
ColE1/pRO1600_*alk*	Default pCom	22.2 ± 1.2	50.6 ± 1.9	439.4 ± 40.4
ColE1/pRO1600_*lac*	Default pCom	35.0 ± 1.9	76.7 ± 11.7	455.6 ± 95.0
ColE1/pRO1600_*tac*	Default pCom	18.5 ± 1.0	42.8 ± 5.7	432.5 ± 81.1
ColE1/pRO1600	Weak	14.0 ± 0.9	25.4 ± 0.3	551.3 ± 29.0
	Moderate	48.4 ± 1.5	78.9 ± 4.2	613.7 ± 51.7
	Strong	4.7 ± 0.3	46.1 ± 9.6	101.5 ± 28.0
RSF1010	Weak	2.7 ± 0.7	11.3 ± 9.8	242.1 ± 266.3
	Moderate	50.7 ± 0.7	67.7 ± 7.6	749.5 ± 94.1
	Strong	49.8 ± 4.6	76.7 ± 11.7	665.5 ± 85.3
ColE1/pRO1600 with terminator	Moderate	42.4 ± 2.4	78.1 ± 11.7	543.3 ± 113.0
	Strong	27.3 ± 2.4	81.1 ± 6.2	336.7 ± 55.1

*^1^CYP concentrations were obtained by CO difference spectroscopy.*

### Translational (RBS) Engineering for the Fine-Tuning of Cyp Expression Levels

**FIGURE 3:**
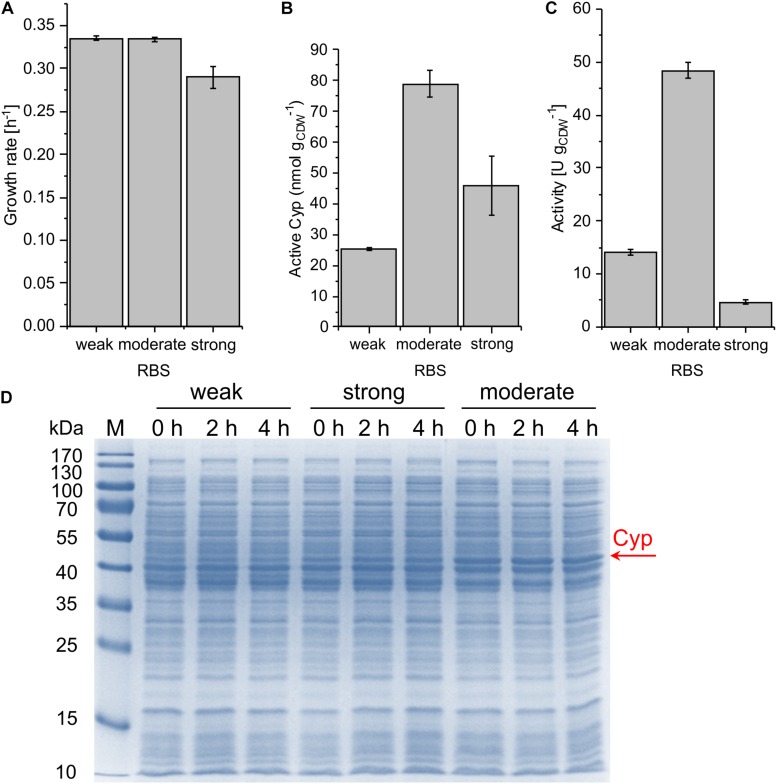
Growth rate **(A)**, concentration of active Cyp determined via CO difference spectra **(B)**, and specific cyclohexane hydroxylation activity **(C)** of *P. taiwanensis* VLB120 pSEVA244_BB32_Cyp (weak RBS), pSEVA244_RBS^∗^_Cyp (moderate RBS), and pSEVA244_BB34_Cyp (strong RBS) after 4 h of induction. Resting cell assays were performed as described in Figure 2 with biomass concentrations of 0.2 and 0.5 g L**^–^**^1^. The bars represent average values. Standard deviations of two independent biological replicates are given. The average experimental errors over all measurements for the growth rate, active Cyp concentration, and activity are 2.0, 9.2, and 4.6%, respectively. SDS-PAGE analyses **(D)** of the strains used for activity assays show the corresponding Cyp content (at 47.4 kDa).

The engineering of the RBS constitutes another strategy to maximize expression levels and is considered a practical approach because of the small number of bases that need to be altered to achieve a wide range of expression levels (Jeschek et al., 2016). With increasing RBS strength, the protein synthesis rate is enhanced (Peretti and Bailey, 1987) in the sense of a more frequent initiation of mRNA translation. The stronger the RBS, the more ribosomes are recruited to this particular site. The RBS sequence is complementary to the 3′ end of the 16S rRNA, which is identical in *Escherichia coli* and *P. taiwanensis* VLB120. We hypothesized that the relative strength of given RBSs is comparable in both strains and thus relied on the readily available expression data for *E. coli*. Three different RBSs with low (BBa_B0032), moderate (RBS^∗^), and high (BBa_B0034) strength were selected (Elowitz and Leibler, 2000; Weiss et al., 2004; Heidorn et al., 2011) based on the Registry of Standard Biological Parts, n.d.). The vector pSEVA244 containing a Kanamycin resistance gene, the pRO1600/ColE1 origin of replication, and the LacI^q^*-P_*trc*_* regulation system from the SEVA platform (Silva-Rocha et al., 2013) was utilized as the basis for further cloning. For all constructs, the same BioBrick scars were introduced between promoter and RBS and between RBS and start codon so that they only differ in their respective RBS sequence.
With the chosen SEVA bricks, pCom10-related issues regarding growth inhibition could successfully be overcome (Figure 3A). The highest specific whole-cell activity of 48.4 ± 1.5 U g_CDW_^–1^ was observed with the moderately strong RBS, coinciding with the highest Cyp amount on the SDS gel, the highest amount of active Cyp as determined via CO difference spectra (Figure 3 and Supplementary Table S2), and the highest turnover number (Table 2). The weakest RBS resulted in a specific activity of 14.0 ± 0.6 U g_CDW_^–1^, coming along with a faint Cyp band on the SDS gel and a turnover number to that obtained with the moderate RBS. The strongest RBS resulted in a low specific activity of 4.7 ± 0.3 U g_CDW_^–1^ and a low turnover number for the Cyp (Table 2). Thereby, the Cyp band on the SDS-PAGE gel was stronger and the CO difference spectra-based Cyp concentration was higher compared to the weak RBS (Figure 3D). Furthermore, the slightly reduced growth rate of the respective strain compared to the other two strains indicates that expression via the strongest RBS hampers cell physiology (Figure 3A). Obviously, most of the translated Cyp enzyme was inactive because of incorrect folding, possibly involving fast enzyme degradation. Further, its activity may have been limited by the NADH supply via the stressed cell metabolism. These results emphasize that the translation initiation rate needs to be optimized so that cell physiology can cope with additional (stress-related) demands, and to enable correct protein folding.

All strains showed a high level of leakiness. Induction did not have any effect on the Cyp amount (Figure 3D). This indicates that the regulation system applied, in combination with the low plasmid copy number of 13 per cell (determined for *Pseudomonas aeruginosa*) (Farinha and Kropinski, 1990), conveys a remarkable basal translation, irrespective of the presence of *lacI*^*q*^ encoding a strong repressor of the *lac* system.

### Gene Dosage Variation for the Optimization of Cyp Gene Expression

The variation of the gene copy number constitutes another common strategy to alter the expression level and typically is realized by utilizing plasmids with different copy numbers per cell. For comparison, the ColE1/pRO1600 origins of replication used in the previous experiments (13 copies per cell in *P. aeruginosa*) (Farinha and Kropinski, 1990) were replaced by the broad-host range replication origin RSF1010. Respective copy numbers are high and mostly host-independent (Meyer, 2009), with 130 ± 40 copies reported for *P. putida* KT2440 (Cook et al., 2018). The RSF1010 origin of replication was tested in combination with all three RBSs, measuring growth rate, Cyp content, and whole-cell activity (Supplementary Table S2).

**FIGURE 4:**
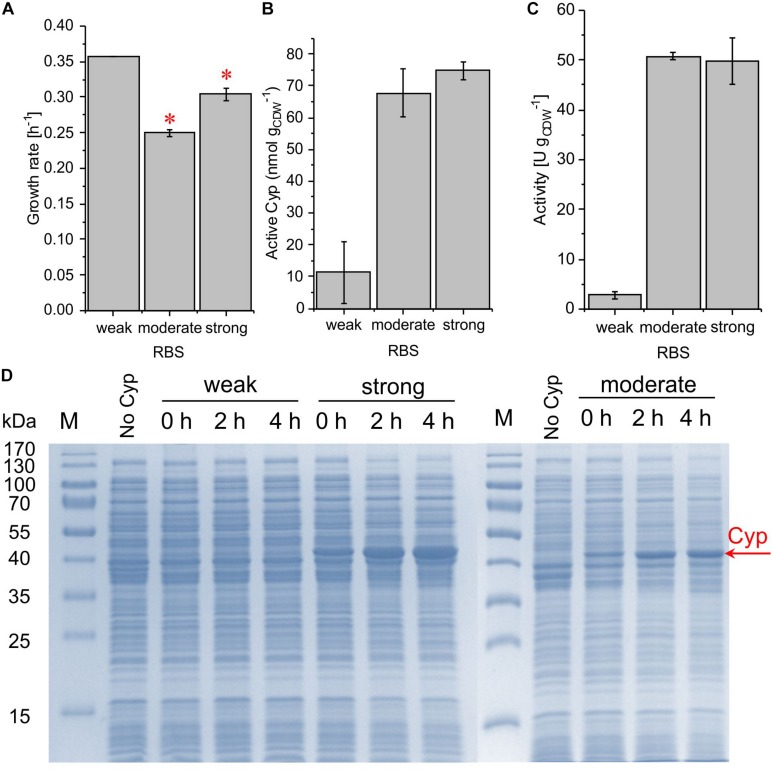
Growth rate **(A)**, concentration of active Cyp determined via CO difference spectra **(B)**, and specific cyclohexane hydroxylation activity **(C)** of *P. taiwanensis* VLB120 pSEVA254_BB32_Cyp (weak RBS), pSEVA254_RBS^∗^_Cyp (moderate RBS), and pSEVA254_BB34_Cyp (strong RBS) after 4 h of induction. (^∗^) Exponential growth only until 1 h after induction, followed by a growth rate decrease. For resting cell assays, cells were processed as described in the legend of Figure 3. The bars represent average values. Standard deviations of two independent biological replicates are given. The average experimental errors over all measurements for the growth rate, active Cyp concentration, and activity are 7.1, 33.6, and 11.5%, respectively. The high overall error of Cyp concentrations is biased by the low value and big error obtained for the weak RBS. SDS-PAGE analyses **(D)** of the strains used for activity assays show the corresponding Cyp content (at 47.4 kDa).

The introduction of the RSF1010 origin was found to strongly influence all these parameters. Its combination with the weakest RBS led to the highest growth rate but involved a low whole-cell activity of 2.7 ± 0.7 U g_CDW_^–1^ and no Cyp detection via SDS-PAGE (Figure 4). The combination of the RSF1010 origin with both the moderate and the strong RBS reduced the growth rate, which only was exponential until 1 h after induction (see Supplementary Figure S3). The final biomass concentration after 4 h of induction was only 0.2 g_CDW_ L^–1^. However, specific whole-cell activities reached high levels of 50.7 ± 0.7 and 49.8 ± 4.6 U g_CDW_^–1^ with the moderate and the strong RBS, respectively (Figure 4C). A much stronger Cyp band was detected for the strong RBS (Figure 4D), whereas, according to CO difference spectra and turnover numbers, active Cyp concentrations were similar with these two RBSs. This indicates that, with the strong RBS, a large Cyp fraction was not appropriately processed (folding, heme incorporation, Figure 4B and Table 2). For both replication systems, the Cyp amount obtained with the moderate RBS is considered appropriate. Again, a strong leakiness was observed. The hampered growth with the high-copy plasmids (RSF1010) indicates that respective constructs and expression impose a significant metabolic burden on the cells. These results emphasize that the origin of replication is a decisive factor for stable expression and biotransformation.

### Introduction of a Terminator Eliminates Leakiness While Preserving the High Specific Activity and Wildtype-Like Growth Physiology

**FIGURE 5:**
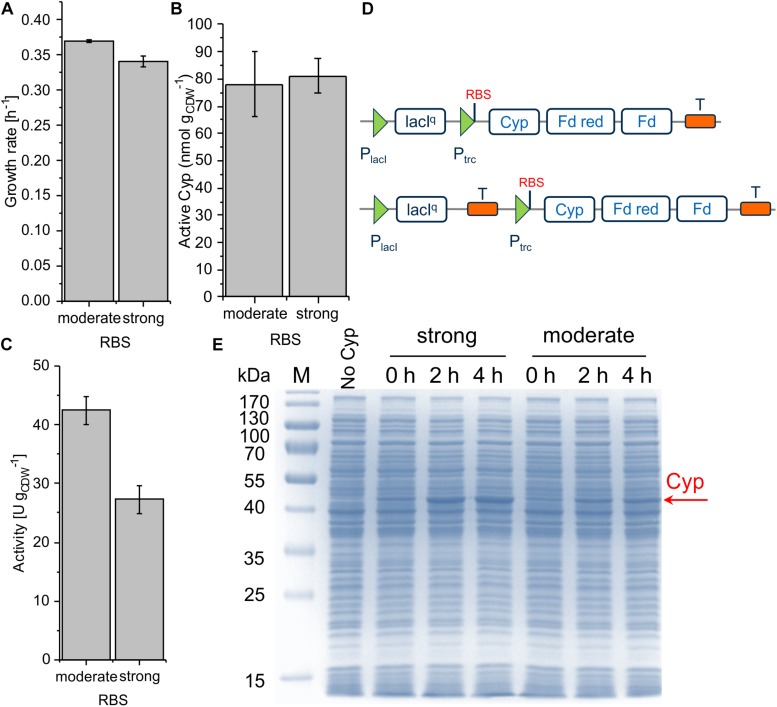
Growth rate **(A)**, concentration of active Cyp determined via CO difference spectra **(B)** and specific cyclohexane hydroxylation activity **(C)** of *P. taiwanensis* VLB120 pSEVA244_T_RBS^∗^_Cyp (moderate RBS) and pSEVA244_T_BB34_Cyp (strong RBS) after 4 h of induction. For resting cell assays, cells were processed as described in the legend of Figure 3. The bars represent average values. Panel **D** illustrates the molecular insertion of the double terminator. Standard deviations of two independent biological replicates are given. The average experimental errors over all measurements for the growth rate, active Cyp concentration, and activity are 1.4, 11.4, and 7.2%, respectively. SDS-PAGE analyses of each strain used for activity assays **(E)** show the corresponding Cyp content (at 47.4 kDa).

The ColE1/pRO1600 system can be considered preferable for Cyp gene expression weighing up all parameters, growth/cell physiology, Cyp expression level, and specific whole-cell activity (Table 2). However, overcoming the leakiness of the construct is necessary to gain appropriate process control. A possible read-through of the Cyp genes from the *lacI*^*q*^ promoter was hypothesized to be the reason for the leakiness. To avoid such a read-through and thus leakiness, a double terminator was introduced after the *lacI*^*q*^ gene (Figure 5D). Following this approach, we investigated moderate and strong RBSs as they enabled reasonable Cyp gene expression in the constructs tested before (Figure 3D). While the transformation of RSF1010 based constructs into *P. taiwanensis* VLB120 was not successful, both ColE1/pRO1600-based constructs exhibited a remarkably enhanced tightness. With the moderate RBS, a high Cyp amount, whole-cell activity, and turnover number were obtained (Figure 5B, Table 2 and Supplementary Table S2). The strong RBS led to higher Cyp expression according to SDS-PAGE, but similar amounts of active Cyp according to CO difference spectra, again indicating non-appropriate Cyp processing (Figures 5B,E). This was further corroborated by the lower specific whole-cell activity obtained with the strong RBS compared to the moderate RBS (Figure 5C). Furthermore, the higher growth rate obtained with the moderate RBS (Figure 5A) indicates a lower metabolic burden for the cells.

The evaluation of induction kinetics with this improved construct revealed a maximal activity of 55.6 ± 2.4 U g_CDW_^–1^ after 6 h of induction (see Supplementary Figure S4), with a slight decrease afterward. To conclude, the improved cyclohexanol producing strain shows fivefold increased activity compared to the non-induced state and exhibits the highest specific whole-cell activity obtained so far with this Cytochrome P450 monooxygenase.

### Catalytic Performance of pSEVA_Cyp and pCom10_Cyp Containing Strains

To evaluate the biocatalytic performance of cells containing the optimized pSEVA_Cyp beyond their initial specific activity, comparative biotransformations of 5 mM cyclohexane were evaluated for 3 h in tightly closed flasks with 1.5 g_CDW_ L^–1^ of cells containing the improved pSEVA_Cyp or pCom10_Cyp (Supplementary Figure S5 and Supplementary Table S3). As expected, the improvement in the Cyp expression level achieved with the pSEVA_Cyp system resulted in a 1.9-fold higher molar cyclohexane conversion yield as compared to the pCom10_Cyp system, whereas the Cyp-related TTN was estimated to be slightly lower with the pSEVA_Cyp system (Table 3). Whereas the selectivity for KA oil formation was 100% in both cases as cyclohexanol and the overoxidation product cyclohexanone were the only reaction products, the pSEVA_Cyp and pCom_Cyp systems gave rise to 89 and 96% cyclohexanol, respectively. The lower TTN and selectivity at higher cyclohexane conversion yields with the pSEVA_Cyp system can be explained by kinetic constraints involving reinforced competition of cyclohexane and cyclohexanol for the active site at low cyclohexane concentrations as a result of gas-liquid mass transfer limitation and product inhibition, finally leading to enhanced cyclohexanol overoxidation. The overall product yield on biomass was improved 1.7-fold with the pSEVA_Cyp system. As the next steps, a detailed characterization of whole-cell biocatalyst kinetics and a suitable feeding strategy for volatile cyclohexane will pave the way for the design of an efficient cyclohexane oxidation process.

**TABLE 3:** Comparison of molar conversion yield, selectivity, total turnover number (TTN), and yield on catalyst for cyclohexane hydroxylation using pCom10_Cyp and pSEVA_Cyp.

	**pCom_Cyp**	**pSEVA_Cyp**
Biotransformation time	3 h	3 h
Cyclohexane conversion [%]	44.5 ± 1.2	82.5 ± 1.1
Selectivity for cyclohexanol (for KA oil) [%]	96.1 ± 0.4(100)	89.3 ± 0.6(100)
Active Cyp concentration [nmol g_CDW_**^–^**^1^]	63.3 ± 7.7	131.9 ± 4.2
TTN [mol_cyclohexanol_ mol_*Cyp*_**^–^**^1^]	22711 ± 3288	18628 ± 484
Yield [mmol_cyclohexanol_ g_CDW_**^–^**^1^]	1.42 ± 0.03	2.46 ± 0.01

## Discussion

Cytochrome P450 monooxygenases (Cyps) are capable of catalyzing a wide range of synthetically challenging hydroxylation, epoxidation, dealkylation, and sulfoxidation reactions for the production of fine chemicals, fragrances, and pharmaceutically active compounds (Urlacher and Schmid, 2006). Although the substantial synthetic potential of Cyps has fiercely triggered respective research, Cyp applications on the industrial scale remained limited to few examples for fine chemicals and pharmaceuticals with high-value gain (Julsing et al., 2008). Limited stability, low activity, their multi-component nature, narrow substrate specificity, cofactor requirements, and the dependency on an electron source constitute the major challenges for the technical application of Cyps, especially for the million ton range production of low-priced chemicals (Bernhardt, 2006; Lundemo and Woodley, 2015), as it is the case for cyclohexanol (Schuchardt et al., 1993; Weissermel and Arpe, 2003). The Cyp employed in this study has been isolated from *Acidovorax* sp. CHX100 and successfully heterologously expressed in *Pseudomonas* and Cyanobacteria (Karande et al., 2016; Hoschek et al., 2019). This study illustrates how holistic genetic engineering can improve the production organism.

### Genetic Engineering to Improve Expression and Activity of (Heterologous) Production Pathways

Transcriptional engineering is the most common strategy to improve expression levels (Jeschek et al., 2017). The promoter sequence itself can be engineered, which has been successfully applied for the production of the secondary metabolites lycopene (Alper et al., 2005) and violacein (Xu et al., 2017), where the product titer could be increased up to threefold in *E. coli*. Similarly, translational engineering (i.e., RBS engineering) was successfully performed to increase the lycopene titer fivefold in *E. coli*. Mostly, these approaches aim at balancing the expression of different genes within a production pathway. Typically, it becomes especially important to optimize the expression level of single enzymes that govern the pathway flux (Xu et al., 2017). Oxygenases often catalyze such rate-limiting steps (Lundemo and Woodley, 2015). Lindmeyer et al. (2015) compared the *alk* regulatory system from *P. putida* GPo1 and the *lac*-system for styrene monooxygenase gene expression in different *P. putida* strains and found that the specific styrene epoxidation activities varied 1.3- to almost fivefold for *P. putida* KT2440 and *P. putida* DOT-TIE, respectively. A similar effect was observed in the present study, where the exchange of the regulation system could double the cyclohexane hydroxylation activity (Figure 2). However, the increase in activity was associated with impaired growth, indicating a negative effect of high level Cyp gene expression on cell physiology, which can be expected to affect whole-cell biocatalyst performance and stability. Our study of different RBS strengths demonstrates that the mere increase in protein amount by accelerating translation is not always associated with higher whole-cell activities (Figure 3). The strongest RBS led to reduced amounts of active Cyp and very low activities, which emphasizes that the achievable Cyp activity does not only depend on the enzyme amount produced, but also on other factors such as the incorporation of the heme group, association with redox partners, and uncoupling. A moderately strong RBS constituted a compromise between high level expression and cell functionality and enabled a two-fold increase in activity compared to the original pCom10_Cyp construct with still acceptable effects on cell physiology.

Apart from the promoter and RBS engineering, the gene copy number, which can be modulated via the plasmid copy number, has been found to strongly influence the achievable enzyme level in *E. coli* (Jahn et al., 2016). By expressing different modules of a Cyp-involving biosynthetic pathway from distinct plasmids in *E. coli*, taxadiene-5a-ol production titers were increased 2400-fold (Ajikumar et al., 2010). However, the maintenance of plasmids, especially those with high copy numbers, poses a high metabolic burden on the cells, which is reflected by reduced growth rates and yields on energy and carbon source (Diaz Ricci and Hernández, 2000). Respective observations also were made in this study. The combination of the high copy origin RSF1010 and the two stronger RBSs severely affected the growth of *P. taiwanensis* VLB120 upon Cyp gene expression (Figure 4). Although an activity of 50 U g_CDW_^–1^ was obtained, a significant fraction of the produced protein appeared to be catalytically inactive. Overexpression can have severe consequences for the host as it can change the lipid composition, reduce the growth rate, and affect the strain’s genetic stability (Nieboer et al., 1993; Chen et al., 1996). Additionally, oxygenase-specific issues such as uncoupling leading to the formation of reactive oxygen species can reduce metabolic activity and cell viability in general (Kadisch et al., 2017).

In this study, the systematic combination of transcriptional, translational, and gene copy number engineering strategies finally enabled a 2.5-fold improvement of the specific hydroxylation activity of recombinant *P. taiwanensis* VLB120 while maintaining cellular fitness.

### Comparison to Other Biocatalytic Cyclohexane Hydroxylation Approaches

Cyclohexane hydroxylation is of high industrial interest, but only a few datasets for biocatalytic processes are currently available (Table 4). What they all have in common is the operation at moderate temperature and ambient pressure, which is an advantage compared to the running industrial process requiring 413–453 K and 7–20 atm (Fischer et al., 2010). The whole-cell-based approach presented in this study reached a conversion of 82.5% with a selectivity of 100% for KA oil and thus outcompeted the chemical catalyst with 8% conversion and 80% selectivity (Fischer et al., 2010). Cyclohexane hydroxylation approaches with isolated monooxygenases suffer from low product formation rates or were achieved by adding different additives (Jiang et al., 1993; Bordeaux et al., 2011; Kawakami et al., 2011). The highest specific turnover number of 3910 mol min^–1^ mol^–1^ has been reported for a modified P450 BM3 in isolated form, but was based on the spectrophotometric determination of NADPH depletion only and thus may include significant uncoupling (Glieder et al., 2002). Besides this high value for P450 BM3, the *P. taiwanensis* VLB120 strains presented in this study showed the highest oxygenase-specific turnover rates reported so far. Whereas data on biocatalytic stability are mostly missing in previous studies, reasonable TTNs could be achieved here. These were 1000-fold higher than those obtained by Bordeaux et al. (2011) for modified CYP153A13a, indicating that the *Acidovorax* Cyp was quite well stabilized within the cellular context. Hoschek et al. (2019) set a benchmark for the utilization of the *Acidovorax* Cyp by applying it in *Synechocystis* sp. 6803 and making use of a biphasic system in a stirred-tank bioreactor with DINP as an organic phase. A specific yield of 49 mmol cyclohexanol per g cell dry weight was reached after an operation time of 52 h, once more demonstrating the potential of whole-cell biocatalysis and this enzyme. For *Synechocystis* as host strain, the achievement of high cell densities combined with sufficient light supply will be a major challenge. Besides this, the overall higher protein amounts in heterotrophic organisms still constitute a significant advantage over Cyanobacteria (Hoschek et al., 2019). The specific yields obtained in this study were lower as only 5 mM cyclohexane were provided as substrate, which was almost depleted at the end of the biotransformation with *P. taiwanensis* VLB120 pSEVA_Cyp as biocatalyst. Consequently, as a next step, the developed biocatalyst has to be investigated in a process setup enabling longer running times and continuous cyclohexane supply as it has been done for *Synechocystis* sp. 6803. Previously, it has been shown that *P. taiwanensis* VLB120 allows for bioreactor operation at high cell densities up to 40 g_biomass_ L^–1^ in a two-liquid phase bioreactor setup (Kuhn et al., 2012).

**TABLE 4:** Literature data for cyclohexane hydroxylations.

**Enzyme**	**Catalyst format**	**additives**	**Temperature (°C)**	**Reaction time**	**Product formation rate (mol min^–1^ mol^–1^)**	**Total turnover number (-)**	**Initial activity (U g_CDW_^–1^)**	**Specific yield (mmol g_CDW_^–1^)**	**References**
sMMOH	Purified enzyme	H_2_O_2_	45	10 min	0.00014	–	–	–	Jiang et al., 1993
P450 BM3	Purified enzyme	–	RT	initial	151	–	–	–	Glieder et al., 2002
P450 BM3	Purified enzyme	PFC9^a^	25	10 min	108	–	–	–	Kawakami et al., 2011
Modified P450 BM3	Purified enzyme	–	RT	initial	3910^b^	–	–	–	Glieder et al., 2002
Modified CYP153A13a	Purified enzyme	–	RT	2 h	3	20	–	–	Bordeaux et al., 2011
*P.taiwanensis* VLB120 pCom10_Cyp	Whole cells	–	30	3 h	336^c^	22 711	20^c^	1.42^d^	This study
*P.taiwanensis* VLB120 pSEVA_Cyp	Whole cells	–	30	3 h	543^c^	18 628	55^c^	2.46^d^	This study
Syn6803_CYP	Whole cells	DINP^e^	30	52 h	–	–	35^c^	49	Hoschek et al., 2019

*^*a*^Perfluorocarboxylic acid with nine carbon atoms acts as dummy substrate to provide space for cyclohexane because of the incomplete occupation of the heme cavity. ^*b*^Calculated from photospectrometrically recorded NADPH depletion including possible uncoupling. ^*c*^Data for the first 10 min of the reaction. ^*d*^Addition of 5 mM cyclohexane as substrate. ^*e*^Diisononyl phthalate added as an organic phase.*

### Future Perspectives

This study demonstrates that *P. taiwanensis* VLB120 is a suitable host for Cyp gene expression and the hydroxylation of alkanes. Besides its solvent-tolerance (Volmer et al., 2017), a large variety of genetic tools are now available for such *Pseudomonas* strains (Martínez-García et al., 2015). Additionally, due to their intrinsic ability to efficiently synthesize heme, the addition of a heme precursor, as it typically is necessary for *E. coli*, is not required. Until now, systematic improvement of whole-cell biocatalysts regarding specific Cyp activity mostly has been missing. Instead, protein engineering and directed evolution mainly with the camphor 5-monooxygenase P450Cam (CYP101) and P450 BM-3 (CYP102A1) have been successful in terms of improved thermostability, broader substrate spectrum, and higher turnover numbers (Appel et al., 2001; Farinas et al., 2001). This study presents an integrated and holistic genetic engineering approach toward optimal, but not necessarily maximal Cyp gene expression, in order to achieve high oxygenation yields on the substrate, biocatalyst, space, and time. This approach is necessary to meet the demands for the development of economically viable processes based on Cyps (Van Beilen et al., 2003; Lundemo and Woodley, 2015).

The improved strain *P. taiwanensis* VLB120 pSEVA_Cyp can now be used as a starting point for the development of strains able to synthesize polymer building blocks such as ε-caprolactone, 6-aminohexanoic acid, or adipic acid. For ε-caprolactone synthesis, the proof of concept has already been shown by Karande et al. (2018). In this case, Cyp was the rate-limiting enzyme so that an improved Cyp activity, as demonstrated here, constitutes an important basis for an increased overall activity of the cascade.

## Conclusion

In this study, we investigated Cyp gene expression and catalysis in recombinant *P. taiwanensis* VLB120 with respect to growth of the respective strain, expression levels, active Cyp amount, and specific whole-cell activities for cyclohexane hydroxylation. Recombinant *P. taiwanensis* VLB120 was systematically engineered on transcriptional, translational, as well as gene dosage levels. Thereby, a remarkable specific whole-cell biocatalyst activity of 55 U g_CDW_^–1^ was achieved. A compromise between expression strength and preserved enzyme functionality and cellular fitness was found to be crucial to come up with high whole-cell activities. Besides this, the optimized strain *P. taiwanensis* VLB120 pSEVA_Cyp also showed high yields on the substrate cyclohexane and biomass. This biocatalyst shows the highest whole-cell activity for cyclohexane hydroxylation reported so far.

## Data Availability Statement

The datasets generated for this study are available on request to the corresponding author.

## Author Contributions

LS and BB designed the research. LS collected and analyzed the data. LS, RK, and BB wrote the manuscript.

## Conflict of Interest

The authors declare that the research was conducted in the absence of any commercial or financial relationships that could be construed as a potential conflict of interest.
