# Supplementary material for: Maximizing Biocatalytic Cyclohexane Hydroxylation by Modulating Cytochrome P450 Monooxygenase Expression in P. taiwanensis VLB120
Source: Front Bioeng Biotechnol. 2020 Feb 27;8:140. doi: 10.3389/fbioe.2020.00140 (PMC7056670; doi:10.3389/fbioe.2020.00140)
Supplement: Supplementary file 1 [file Data_Sheet_1.PDF]

Supplementary Material

1 Molecular biology methods

For the construction of plasmids pCom_lac _Cyp and pCom_tac_Cyp, the monooxygenase, ferredoxin and ferredoxin reductase genes were isolated from the plasmid pCapro (Karande et al., 2018) by restriction with NdeI and AscI. This purified fragment was ligated into pCom10_lac (Lindmeyer et al., 2015b)or pCom10_tac (Lindmeyer et al., 2015a) cut with the same enzymes, respectively.

To generate the Cyp expression vectors with different RBSs and copy numbers, pSEVA244 and pSEVA254 (Jahn et al., 2016) were employed containing a ColE1/pRO1600 or RSF1010 origin of replication and digested with XmaJI. Cyp, ferredoxin and ferredoxin reductase were amplified from pCapro with the primers listed in Table 1 (PLS005/7/8 and PLS006). The purified fragment and the vector backbone were fused by Gibson assembly.

The double terminator was amplified from the vector pSB1AC3_Ptrc1O_GFPmut3b (Huang et al., 2010) using primers PLS009 and PLS010 (Tab. S2). A PCR with the vector pSEVA244 was conducted with primers PLS011 and PLS012 and both fragments were brought together via Gibson assembly. The Cyp genes were inserted afterwards as described above.

Supplementary Table S1: Primer used during the cloning. **binding region**, overlap to vector, scar, RBS

Primer#	Function	Sequence
PLS005	CYP fwd, BBa_B0032	TGTGAGCGGATAACAATTTCACACCTAGGAGAGTCACACAGGAAAGTACTAG**ATGACTCAGACTGCTGCGGC**
PLS006	CYP rev	GAGCTCGAATTCGCGCGGCCGCGGCCTAGG**TCAGTGCTGCCCTTGCG**
PLS007	CYP fwd, BBa_B0034	TGTGAGCGGATAACAATTTCACACCTAGGAGAGAAAGAGGAGAAATACTAG**ATGACTCAGACTGCTGCGGC**
PLS008	CYP fwd, RBS*	TGTGAGCGGATAACAATTTCACACCTAGGAGAGTAGTGGAGGTTACTAG**ATGACTCAGACTGCTGCGGC**
PLS009	Term fwd	GATCTGGTTTGACAGCTTATCATCG**CCAGGCATCAAATAAAACG**
PLS010	Term rev	CGCCTTGAGCGACACGAATTATGCA**TATAAACGCAGAAAGGCCC**
PLS011	pSEVA244 fwd	**TGCATAATTCGTGTCGCTC**
PLS012	pSEVA244 rev	**CGATGATAAGCTGTCAAACCAG**

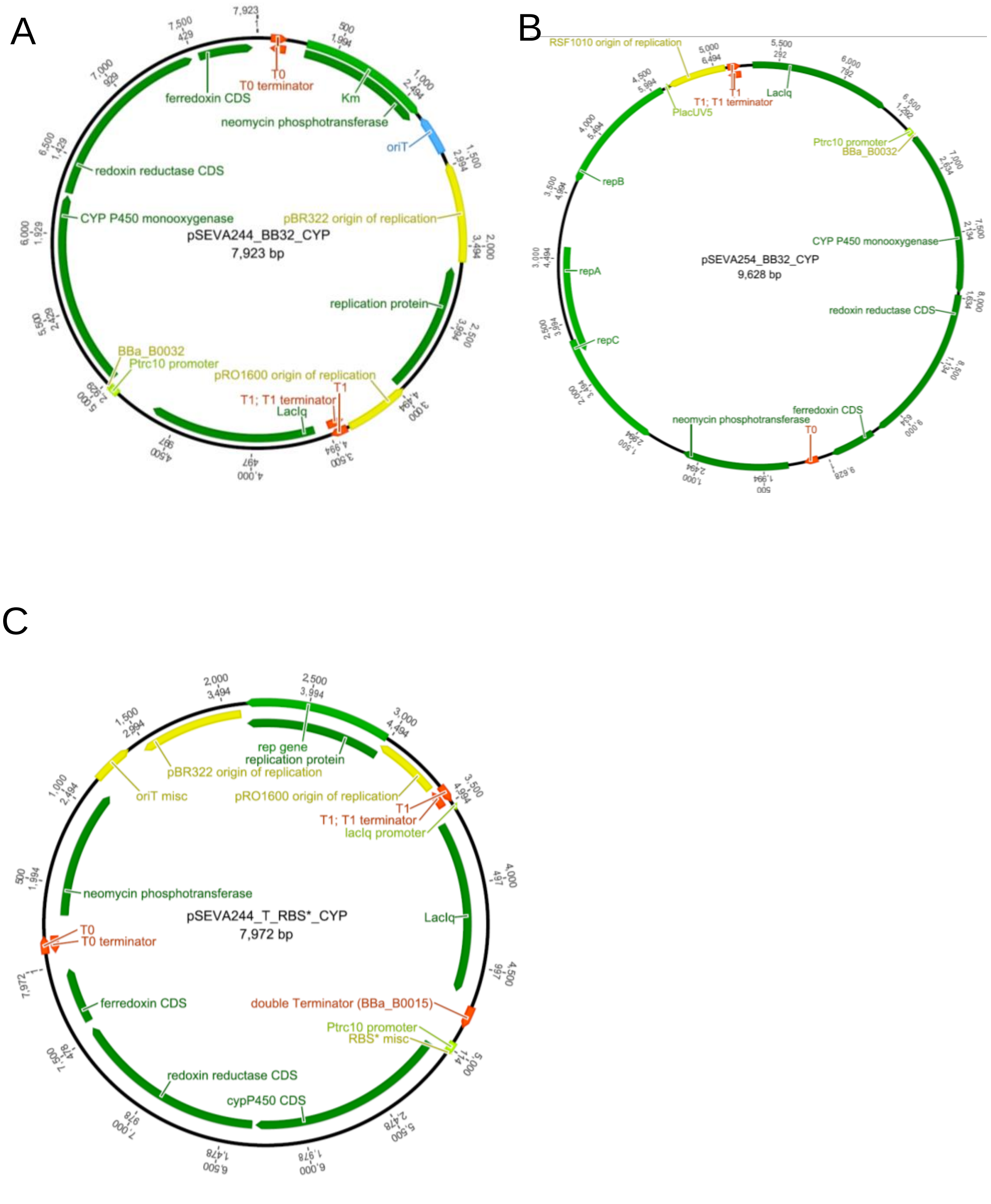

Supplementary Figure S1. Exemplary vector maps for pSEVA244_RBS_Cyp (A) pSEVA254_RBS_Cyp and pSEVA244_T_RBS_Cyp. The vectors are based on the pSEVA collection (Silva-Rocha et al., 2013).

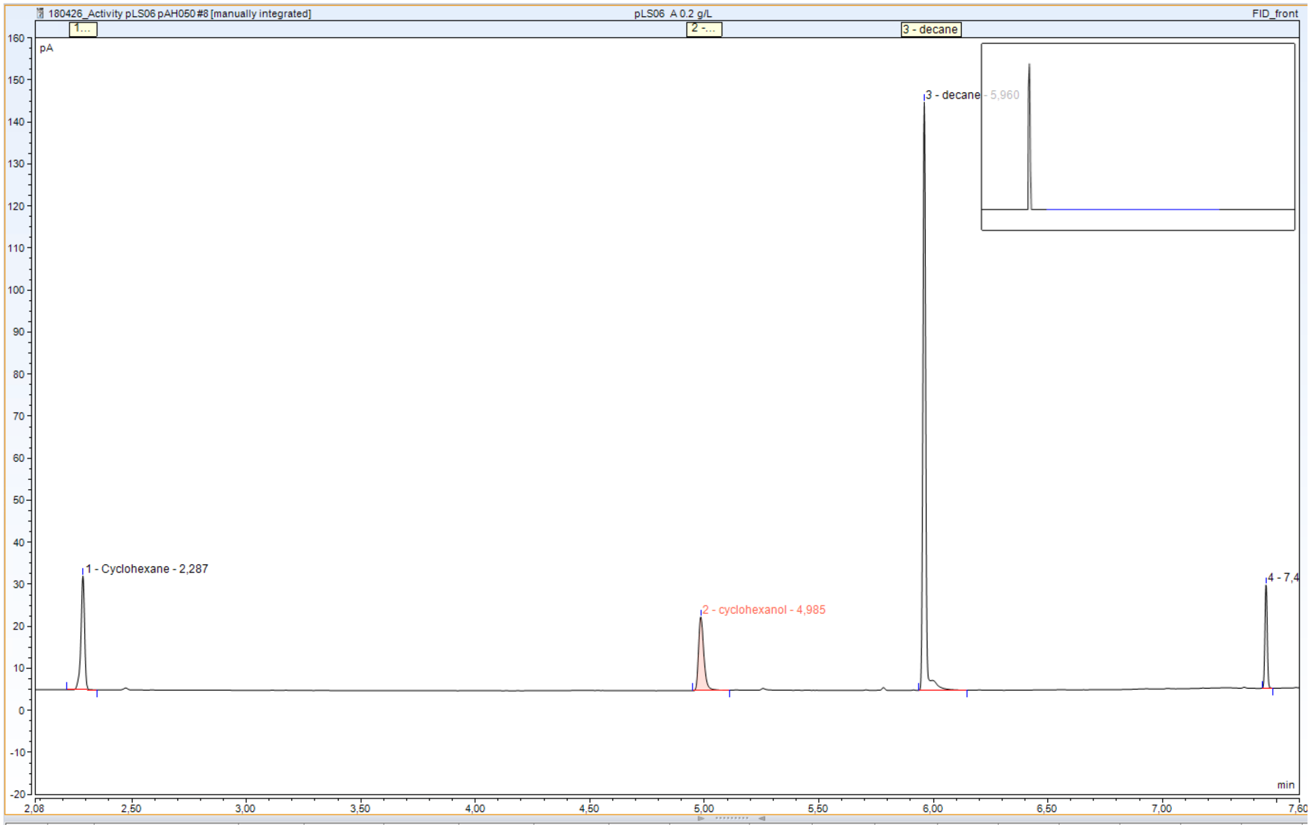

Supplemementary Figure S2: Exemplary GC chromatogram. The GC chromatogram is shown for *P. taiwanensis* VLB120 pSEVA254_BB34_Cyp (see Fig. 4, strong RBS) with the retention times for cyclohexane, cyclohexanol and n-decane of 2.3, 5.0, and 6.0 min, respectively.

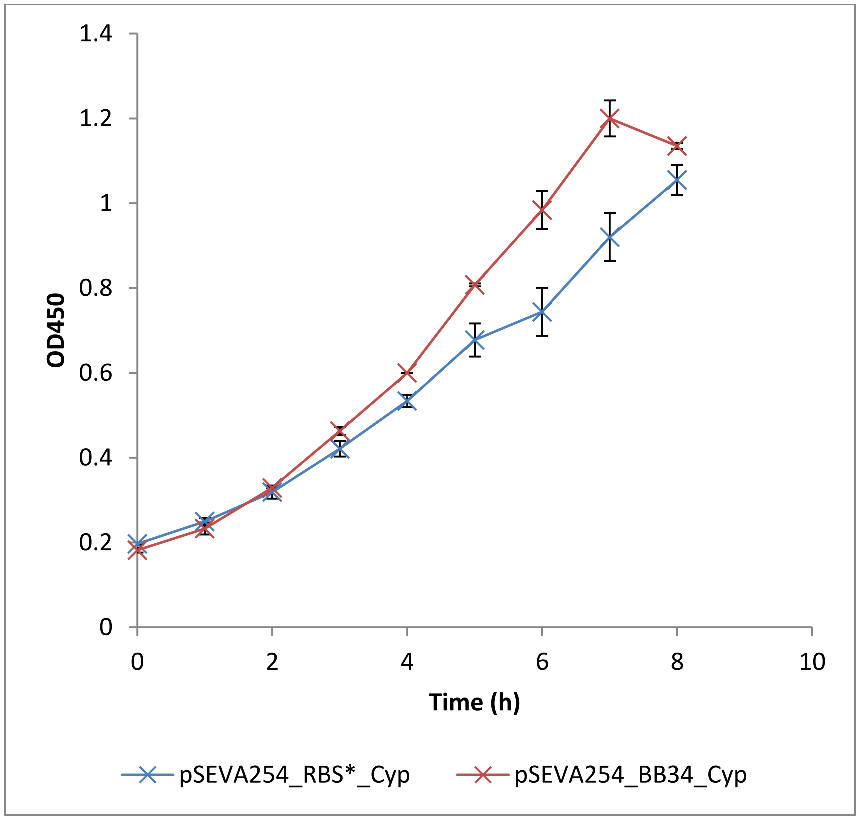

Supplementary Figure S3. Growth curves for *P. taiwanensis* VLB120 pSEVA244_RBS*_Cyp and pSEVA254_BB34_Cyp. The cultures were induced 4 h after inoculation. Cells were cultivated in M9* medium with 0.5 % (w/v) glucose at 30°C, 200 rpm. One OD450 unit corresponds to a biomass concentration of 0.186 g_{CDW} L^{-1} for *P. taiwanensis* VLB120 (Halan et al., 2010).

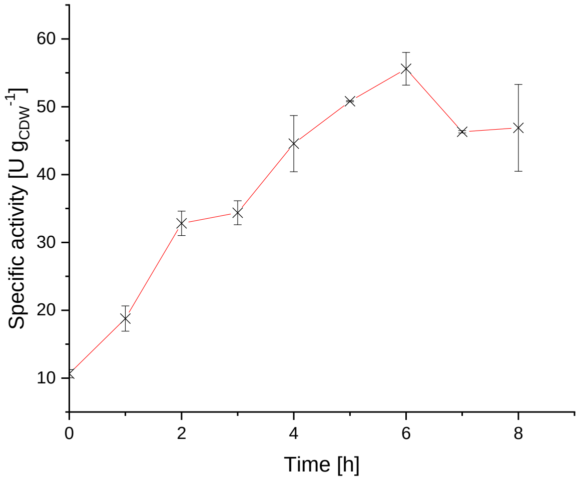

Supplementary Figure S4. Cyclohexane hydroxylation activity *P. taiwanensis* VLB120 pSEVA244_T_RBS*_Cyp (intermediate). Cells were induced 3 h after inoculation and harvested every hour for resting cell assays. For resting cell assays, cells were cultivated in M9* medium with 0.5 % (w/v) glucose and resuspended in Kpi buffer supplemented with 1 % (w/v) respective carbon source to a biomass concentration of 0.2 g/L. Assays were performed with 1 mL of cell suspension in Pyrex tubes in a water bath, at 30°C and 250 rpm. Reactions were started by adding 1.25 µL pure cyclohexane (liquid cyclohexane concentration is equal to 10 µL in flasks 180 µM in aq. phase) and stopped after 10 min by quenching with diethyl ether. The bars represent average values and standard deviations of two independent biological replicates.

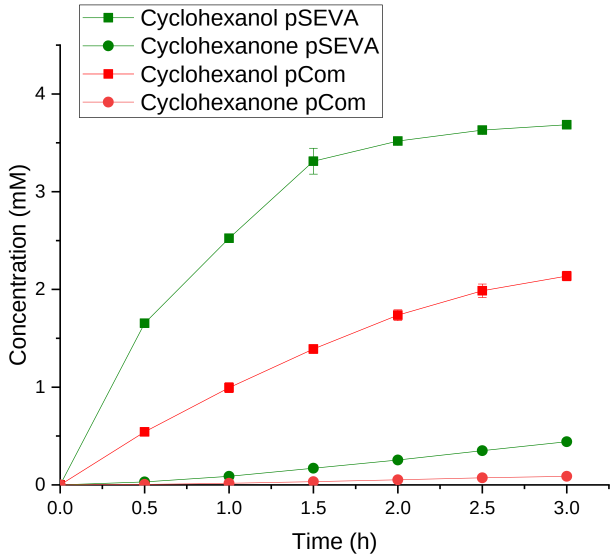

Supplementary Figure S5. Reaction course of the biotransformation employing *P. taiwanensis* VLB120 pCom_Cyp (red) and pSEVA_Cyp (green). Reaction products are cyclohexanol and the overoxidation product cyclohexanone, which are quantified by GC. Reactions were started by adding pure cyclohexane to a total concentration of 5 mM (referred to in the aqueous phase). For further details see Materials and Methods section. The bars represent average values and standard deviations of two independent biological replicates.

Raw data for results presented

Specific activities given in Figs. 2-5 were obtained from GC-derived cyclohexanol concentrations, which were calculated based on calibration curves referring to the internal standard n-decane. Cyclohexanol concentrations, OD_{450}-derived cell concentrations, and activity calculations are given in Table S2. Specific activities were calculated based on the produced cyclohexanol concentration within 10 min taking into account the measured cell concentration in the flask.

Supplementary Table S2: Raw data for Figs. 2-5.

	Construct name	Concentration	Cell concentration	Activity		
		mM	g L^{-1}	U g_{CDW}^{-1}		
		in aqueous phase			Average	Standard deviation
		Cyclohexanol				
Figure 2	pCom10_lac_Cyp A	0.337	0.975	34.5	35.0	1.9
	pCom10_lac_Cyp B	0.355	0.962	36.9		
	pCom10_lac_Cyp A	0.072	0.201	35.9		
	pCom10_lac_Cyp B	0.064	0.199	32.4		
	pCom10_tac_Cyp A	0.184	0.960	19.1	18.5	1.0
	pCom10_tac_Cyp B	0.174	0.973	17.9		
	pCom10_tac_Cyp A	0.033	0.189	17.4		
	pCom10_tac_Cyp B	0.039	0.201	19.6		
	pCom10_Cyp A	0.034	0.149	22.9	22.2	1.2
	pCom10_Cyp B	0.032	0.156	20.4		
	pCom10_Cyp A	0.167	0.740	22.6		
	pCom10_Cyp B	0.171	0.748	22.9		
Figure 3	pSEVA244_BB32_Cyp A	0.024	0.166	14.7	14.0	0.6
	pSEVA244_BB32_Cyp B	0.025	0.175	14.2		
	pSEVA244_BB32_Cyp A	0.058	0.424	13.7		
	pSEVA244_BB32_Cyp B	0.060	0.448	13.4		
	pSEVA244_BB34_Cyp A	0.009	0.193	4.5	4.7	0.3
	pSEVA244_BB34_Cyp B	0.008	0.184	4.5		
	pSEVA244_BB34_Cyp A	0.025	0.478	5.2		
	pSEVA244_BB34_Cyp B	0.021	0.476	4.5		
	pSEVA244_RBS*_Cyp A	0.084	0.179	46.9	48.4	1.5
	pSEVA244_RBS*_Cyp B	0.091	0.182	50.1		
	pSEVA244_RBS*_Cyp A	0.220	0.463	47.5		
	pSEVA244_RBS*_Cyp B	0.223	0.454	49.2		
Figure	pSEVA254_BB32_Cyp A	0.007	0.211	3.3	2.7	0.7
	pSEVA254_BB32_Cyp B	0.004	0.223	1.8		
	pSEVA254_BB32_Cyp A	0.016	0.551	3.0		

	pSEVA254_BB32_Cyp B	0.016	0.526	3.0		
	pSEVA254_BB34_Cyp A	0.114	0.214	54.5	49.8	4.6
	pSEVA254_BB34_Cyp B	0.103	0.196	52.9		
	pSEVA254_BB34_Cyp A	0.164	0.355	46.7		
	pSEVA254_BB34_Cyp B	0.155	0.333	45.1		
	pSEVA254_RBS*_Cyp A	0.113	0.225	50.3	50.7	0.7
	pSEVA254_RBS*_Cyp B	0.113	0.221	50.9		
	pSEVA254_RBS*_Cyp A	0.219	0.424	51.6		
	pSEVA254_RBS*_Cyp B	0.217	0.433	50.0		
Figure 5	pSEVA244_T_BB34_Cyp A	0.145	0.588	24.7	27.3	2.4
	pSEVA244_T_BB34_Cyp B	0.153	0.588	26.0		
	pSEVA244_T_BB34_Cyp C	0.136	0.552	24.6		
	pSEVA244_T_BB34_Cyp D	0.152	0.552	27.5		
	pSEVA244_T_BB34_Cyp A	0.062	0.212	29.1		
	pSEVA244_T_BB34_Cyp B	0.067	0.212	31.8		
	pSEVA244_T_BB34_Cyp C	0.055	0.204	27.0		
	pSEVA244_T_BB34_Cyp D	0.056	0.204	27.6		
	pSEVA244_T_RBS*_Cyp A	0.343	0.565	60.7	42.4	2.4
	pSEVA244_T_RBS*_Cyp B	0.245	0.565	43.4		
	pSEVA244_T_RBS*_Cyp C	0.219	0.497	44.1		
	pSEVA244_T_RBS*_Cyp D	0.222	0.497	44.6		
	pSEVA244_T_RBS*_Cyp A	0.100	0.246	40.5		
	pSEVA244_T_RBS*_Cyp B	0.109	0.246	44.3		
	pSEVA244_T_RBS*_Cyp C	0.097	0.231	42.2		
	pSEVA244_T_RBS*_Cyp D	0.088	0.231	38.0		

The biotransformation data given in Table 3 were obtained from GC-derived cyclohexane, cyclohexanol and cyclohexanone concentrations, which were calculated based on calibration curves referring to the internal standard n-decane. The concentrations are given in Table S3, where cyclohexanol and cyclohexanone are both considered as products of the biotransformation. The data given in Tab. 3 were calculated as described in Materials and Methods after a biotransformation time of 3 h with a cell concentration of 1.5 g L^{-1}.

Supplementary Table 3: Raw data for Table 3 and Supplementary Figure S5.

		**Concentration cyclohexane**			**Concentration cyclohexanol**			**Concentration cyclohexanone**			Sum of Products	
		mM			mM			mM			mM	
Time		in aqueous phase			in aqueous phase			in aqueous phase				
h	Injection Name		Average	Standard deviation		Average	Standard deviation		Average	Standard deviation		
0.5	pCom t1 A	0.056	0.054	0.003	0.515	0.543	0.039	0.004	0.005	0.001	0.548	0.040
0.5	pCom t1 B	0.052			0.571			0.005				
1	pCom t2 A	0.045	0.046	0.001	0.959	0.995	0.052	0.014	0.016	0.003	1.011	0.055
1	pCom t2 B	0.047			1.032			0.019				
1.5	pCom t3 A	0.050	0.050	0.000	1.358	1.390	0.045	0.030	0.033	0.004	1.423	0.048
1.5	pCom t3 B	0.050			1.421			0.035				
2	pCom t4 A	0.040	0.043	0.004	1.698	1.737	0.055	0.048	0.052	0.006	1.789	0.062
2	pCom t4 B	0.046			1.776			0.057				
2.5	pCom t5 A	0.039	0.040	0.001	1.937	1.986	0.069	0.066	0.072	0.009	2.058	0.078
2.5	pCom t5 B	0.041			2.035			0.078				
3	pCom t6 A	0.038	0.037	0.001	2.103	2.137	0.049	0.080	0.087	0.010	2.224	0.059
3	pCom t6 B	0.036			2.172			0.094				
0.5	pSEVA t1 A	0.014	0.015	0.001	1.661	1.654	0.010	0.029	0.031	0.003	1.685	0.013
0.5	pSEVA t1 B	0.016			1.646			0.033				
1	pSEVA t2 A	0.010	0.010	0.001	2.528	2.524	0.006	0.084	0.088	0.006	2.612	0.012
1	pSEVA t2 B	0.009			2.520			0.093				
1.5	pSEVA t3 A	0.008	0.007	0.001	3.406	3.312	0.132	0.166	0.170	0.006	3.482	0.138
1.5	pSEVA t3 B	0.006			3.218			0.175				
2	pSEVA t4 A	0.005	0.005	0.000	3.531	3.518	0.019	0.242	0.255	0.018	3.772	0.037
2	pSEVA t4 B	0.005			3.504			0.267				
2.5	pSEVA t5 A	0.003	0.003	0.000	3.646	3.630	0.023	0.336	0.351	0.021	3.980	0.044
2.5	pSEVA t5 B	0.003			3.613			0.365				
3	pSEVA t6 A	0.003	0.003	0.000	3.670	3.685	0.022	0.419	0.441	0.032	4.126	0.053
3	pSEVA t6 B	0.003			3.700			0.464				

References

Halan, B., Schmid, A., and Buehler, K. (2010). Maximizing the productivity of catalytic biofilms on solid supports in membrane aerated reactors. *Biotechnol. Bioeng.* 106**,** 516-527.

Huang, H.-H., Camsund, D., Lindblad, P., and Heidorn, T. (2010). Design and characterization of molecular tools for a Synthetic Biology approach towards developing cyanobacterial biotechnology. *Nucleic Acids Res.* 38**,** 2577-2593.

Jahn, M., Vorpahl, C., Hübschmann, T., Harms, H., and Müller, S. (2016). Copy number variability of expression plasmids determined by cell sorting and Droplet Digital PCR. *Microb. Cell Fact.* 15**,** 211.

Karande, R., Salamanca, D., Schmid, A., and Buehler, K. (2018). Biocatalytic conversion of cycloalkanes to lactones using an in-vivo cascade in *Pseudomonas taiwanensis* VLB120. *Biotechnol. Bioeng.* 115**,** 312-320.

Lindmeyer, M., Jahn, M., Vorpahl, C., Müller, S., Schmid, A., and Bühler, B. (2015a). Variability in subpopulation formation propagates into biocatalytic variability of engineered Pseudomonas putida strains. *Front. Microbiol.* 6**,** 1042.

Lindmeyer, M., Meyer, D., Kuhn, D., Bühler, B., and Schmid, A. (2015b). Making variability less variable: matching expression system and host for oxygenase-based biotransformations. *J. Ind. Microbiol. Biotechnol.* 42**,** 851-866.

Silva-Rocha, R., Martínez-García, E., Calles, B., Chavarría, M., Arce-Rodríguez, A., De Las Heras, A., Páez-Espino, A.D., Durante-Rodríguez, G., Kim, J., Nikel, P.I., Platero, R., and De Lorenzo, V. (2013). The Standard European Vector Architecture (SEVA): a coherent platform for the analysis and deployment of complex prokaryotic phenotypes. *Nucleic Acids Res.* 41**,** D666-D675.
